# A Retrospective Application of the Arbon and Hartman Models to the Union Cycliste International Mountain Bike World Cup

**DOI:** 10.1017/S1049023X23006222

**Published:** 2023-10

**Authors:** Heather Tucker, Timothy Duncan, Paul A. Craven, Christopher Goode, James Scheidler

**Affiliations:** 1. Emergency Medicine, West Virginia University, Morgantown, West Virginia USA; 2. University of New Mexico, School of Medicine, Albuquerque, New Mexico USA

**Keywords:** mass-gathering event, prehospital medicine, UCI mountain bike World Cup

## Abstract

**Introduction:**

Outdoor activities have accelerated in the past several years. The authors were tasked with providing medical care for the Union Cycliste International (UCI) mountain biking World Cup in Snowshoe, West Virginia (USA) in September 2021. The Hartman and Arbon models were designed to predict patient presentation and hospital transport rates as well as needed medical resources at urban mass-gathering events. However, there is a lack of standardized methods to predict injury, illness, and insult severity at rural mass gatherings.

**Study Objective:**

This study aimed to determine whether the Arbon model would predict, within 10%, the number of patient presentations to be expected and to determine if the event classification provided by the Hartman model would adequately predict resources needed during the event.

**Methods:**

Race data were collected from UCI event officials and injury data were collected from participants at time of presentation for medical care. Predicted presentation and transport rates were calculated using the Arbon model, which was then compared to the actual observed presentation rates. Furthermore, the event classification provided by the Hartman model was compared to the resources utilized during the event.

**Results:**

During the event, 34 patients presented for medical care and eight patients required some level of transport to a medical facility. The Arbon predictive model for the 2021 event yielded 30.3 expected patient presentations. There were 34 total patient presentations during the 2021 race, approximately 11% more than predicted. The Hartman model yielded a score of four. Based on this score, this race would be classified as an “intermediate” event, requiring multiple Advanced Life Support (ALS) and Basic Life Support (BLS) personnel and transport units.

**Conclusion:**

The Arbon model provided a predicted patient presentation rate within reasonable error to allow for effective pre-event planning and resource allocation with only a four patient presentation difference from the actual data. While the Arbon model under-predicted patient presentations, the Hartman model under-estimated resources needed due to the high-risk nature of downhill cycling. The events staffed required physician skills and air medical services to safely care for patients. Further evaluation of rural events will be needed to determine if there is a generalized need for physician presence at smaller events with inherently risky activities, or if this recurring cycling event is an outlier.

## Introduction

The quest to descend steeper and rockier slopes on two wheels, run longer and faster across more varied terrain, jump from greater heights, and summit taller peaks has increased over the past decade. Outdoor adventures have accelerated in the past several years by the need for presumably “safer” activities during the coronavirus disease 2019 (COVID-19) pandemic. According to the 2021 outdoor participation trends report gathered by the Outdoor Industry Association (Boulder, Colorado USA), 53% of Americans aged six years and over participated in outdoor recreation at least once in 2020, which is 7.1 million more Americans than the year prior.^1^ Laskowski-Jones, et al detailed the rapidly increasing popularity of obstacle courses and extreme-challenge outdoor events.^2^ Wilderness, sports, and event medicine come together at these events where race components, geography, participant demographics, risk assessment, and legal elements must be considered to provide effective medical care to participants.^2^

The COVID-19 pandemic appears to have exponentially increased outdoor participation with the need for social distancing and remote work allowing people greater schedule and geographic flexibility. With an increase in participants and spectators in adventure sports, an increase in injuries related to these events can be expected as well. The authors of this study were tasked with providing medical care for the Union Cycliste International (UCI; Aigle, Switzerland) downhill mountain biking World Cup in Snowshoe, West Virginia (USA) in September 2021. The UCI is an international cycling organization that hosts professional bike races in a variety of cycling disciplines, including downhill, cross country, and short-track mountain biking. The authors were charged with organizing and providing medical care and supplies in conjunction with Snowshoe’s bike patrol team for this six-day international event. They were guided by past events - particularly the Snowshoe UCI races held in 2019 - to determine the expected medical needs. This assessment included considering the minimum number of medical providers needed to staff the event, the supplies deemed critical to have on-hand, characteristics of expected injuries, and to what capacity the surrounding hospitals would be able to provide care for the 7,500 expected spectators and participants at the event. In preparation for the event, a literature search revealed the lack of a standardized method to predict injury, illness, and insult severity at rural mass-gathering events.

The most relevant predictive methods used in common practice to aid in preparation for mass-gathering events include the Hartman and Arbon models, which were designed to predict patient presentation and hospital transport rates as well as needed medical resources at urban mass-gathering events.^3,4^ These peer-reviewed methods are designed for much larger urban events, but the authors were unable to identify any validated resources to help predict patient presentation rates at smaller events, similar to the UCI race. This study aimed to determine whether the Arbon model would predict, within 10%, the number of patient presentations to be expected and to determine if the event classification provided by the Hartman model would adequately predict resources needed during the event. If these models are able to accurately predict patient presentations and resource needs, this may help providers preparing for similar events in the future, by ensuring optimal medical care for participants and spectators, providing a comfortable and safe practice environment for providers, helping to prevent local medical system overload, and leading to a successful event overall.

## Methods

This study was designed as an observational study comparing real versus predicted cases and needed medical resources using the Arbon and Hartman models during a downhill-cycling event. This study was designated as non-human subject research and was approved by the West Virginia University Institutional Review Board (Morgantown, West Virginia USA) as protocol number 2108390083 and titled “UCI 2021 - Austere Mass Gathering Event Emergency Medicine.” The primary outcome measured was the number of patient presentations for medical care. Need for referral or transport to a higher level of care was measured as a secondary outcome. Predicted presentation rates were calculated using the Arbon model, which was then compared to the actual observed presentation rates for both the 2019 and 2021 UCI World Cup races at Snowshoe. Predicted hospital transportation rates were also calculated using the formula derived by the Arbon study. Additionally, the event classification provided by the Hartman model was compared to the resources utilized during the event to determine if the Hartman model classification accurately predicted necessary resources.

Two races on the UCI 2021 World Cup circuit were held in Snowshoe, West Virginia. Snowshoe is a ski resort on the eastern border of rural West Virginia, located approximately 30 miles from the nearest medical facility, which is a 25-bed critical access hospital.^5^ In addition, the UCI event lies within the National Radio Quiet Zone limiting communication capabilities.^6^ Race data were collected from UCI event officials, including the total number of participants and spectators. Individual injury data were collected from participants at time of presentation for medical care. A team member with no patient care responsibilities was designated to obtain written informed consent from individuals for this study at time of presentation for care. All patient contacts were unplanned requests for medical treatment.

Data collected included name, age, sex, nationality, time of injury, location of injury, medical history, injury type, need for transport or referral to medical facility, COVID-19 immunization status, alcohol use prior to presentation, injury occurring during practice or race conditions, and whether the injury occurred on the downhill or cross-country track. Patient presentations were cross-referenced with encounter medical documentation obtained by the bike patrol team to ensure both clinical accuracy and complete capture of all encounters.

## Results

Per data provided by UCI staff, approximately 7,500 spectators and participants attended the 2021 event. Of those, 332 were participants. Over the six-day period, 34 patients presented for medical care and eight patients required transport to a medical facility via ambulance, helicopter, or personal vehicle. Of the 34 patients who presented, 24 were participants and ten were spectators, which yielded a presentation rate of 7.2% among participants and 0.1% among spectators. The predicted number of patient encounters using the Arbon models was 30.3. The calculations, based on the Arbon model for event characteristics (Table 1), are shown below.

**Table 1.** Arbon Model for Predicting Patient Presentations^3^

Y = Predicted Patient Presentations Y = −78.184699 + (−31.488567*SEATS) + (84.556898*BOUNDED) + (42.370240*INDOOR) + (81.319501*OUTDOOR) + (−20.390940*SPORT) + (−0.616134*HUMID) + (−0.000456*ATTEND) + (0.000016246*ATTHUMID) + (20.067439*DAY- NIGHT)	
Seats =	1: When event population is non-mobile
	0: When population is mobile
Bounded =	1: When event is fenced/bounded
	0: When event is unbound
Indoor =	1: When event is indoors
	0: When event is outdoors
Outdoor =	1: When event is outdoors
	0: When even is indoors
Sport =	1: For all sporting events
	0: For all non-sporting events
Humid =	Level of humidity expected for day of event
Attendance =	Number of persons expected to attend
ATTHUMID =	Products of multiplying attendance and humidity
Day-Night =	1: When event is both day and night
	0: When event is only day or only night
Seats =	1: When event population is non-mobile

The formula legend in the original Arbon study was used to determine the following variable inputs for the formula, as shown in Table 2.^3^

**Table 2.** Variable Inputs as Determined by the Formula Legend in the Original Arbon Model^3^

Seats =	0: Population as mobile
Bounded =	1: Population and participants were bounded
Indoor =	0: Event was outdoor only
Outdoor =	1: Event was outdoor only
Sport =	1: Event was a sporting event
Humidity =	68% daily average @ 11:50 am
Attendance =	7,500
ATTHUMID =	7,500 x 68
Day-Night =	0: Only daytime event

The Arbon predictive model for the 2021 event yielded 30.3 expected patient presentations. There were 34 total patient presentations during the 2021 event, approximately 11% more than predicted. The same calculation was used for the 2019 race, but with the attendance of that event at 10,000 and average humidity of 55%. For the 2019 event, 37.8 presentations were predicted with a total of 42 actual presentations, a 10% increase.^7^ Characteristics of the presenting patients can be seen in Table 3.

**Table 3.** Demographic and Clinical Characteristics

Characteristic	Value	
Year	2021 (n = 34)	2019 (n = 42)
Gender, n (%)		
Male	18 (52.9)	31 (73.8)
Female	16 (47.1)	11 (26.2)
Participant	24 (70.6)	25 (59.5)
Spectator	10 (29.4)	17 (40.5)
Age in Years, Mean (SD)	28.5 (SD = 14.7)	28.6 (SD = 12.9)
Alcohol Use	3 (8.8)	0 (0.0)
Received COVID-19 Vaccine	23 (67.6)	n/a
Downhill	22 (73.3)	n/a
Cross Country	8 (26.7)	n/a
Required Transfer or Referral	8 (23.5)	9 (21.4)
Private Vehicle	5 (14.7)	7(16.7)
Ambulance	2 (5.9)	1(2.4)
Helicopter	1 (2.9)	1(2.4)
Transfers Avoided	10 (29.4)	

When applied to the variables and attendance of the 2021 UCI World Cup, the Hartman model (Table 4) yielded a score of four, with one point given for each of the following: weather (less than 90 degrees F, but not climate controlled); attendance (between 1,000 and 15,000); alcohol consumption (not significant, but not zero); and crowd intention mixed (both animated and calm). Based on this score, this race would be classified as a “intermediate” event, requiring multiple Advanced Life Support (ALS) and Basic Life Support (BLS) personnel and two transport units.^4^

**Table 4.** The Hartman Model with UCI Event Values Bolded^4^

Variable	Points		
	2	1	0
Weather	>90°F (Heat Index)	**80°F-90°F (Heat Index)**	Climate Controlled Environment
	<0°F (Wind Chill)	**0°F-40°F (Wind Chill)**	
	No Climate Controlled Shelter	**Minimal Climate Controlled Shelter**	
Peak Attendance	>15,000	**1,000-15,000**	<1,000
Ethanol Consumption	Significant	**Limited**	None
Crowd Age	Older	Mixed	**Younger**
Crowd Intent	Animated/Rowdy	**Intermediate**	Calm

A special event and mass-gathering medical care planning guide was released by the state of Minnesota (USA) in 2015 along with a model like the Hartman model, but with one additional variable: transport time to a hospital. In this model, two points are given if transport is greater than 30 minutes, one point given is transport is from 20-29 minutes, and no points if the transport takes less than 20 minutes.^8,9^ The expanded model classifies high risk events as those with total scores greater than or equal to five, or scores of two in two different categories. Intermediate risk events require BLS and ALS on site with aid stations and lower risk events should have BLS on site. With this model, the 2021 UCI event would have been given a score of six and classified as “high risk,” requiring ALS on site, roaming teams of ALS personnel, and ideally, a physician on site.

## Discussion

In 2001, Arbon, Bridgewater, and Smith created a model to predict rates of patient presentation and transportation to hospitals for mass-gathering events, based on data collected from large, public events in Australia. They accounted for factors including crowd size, temperature, humidity, alcohol, event boundaries, personnel on duty, and venue type. They studied 201 mass gatherings over 12 months with a combined audience of 12 million people and sample size of 11,956 patient presentations, followed by derivation of a regression model to predict presentation rates at future events. They defined mass-gathering events as those with greater than 1,000 people in attendance, but used those with greater than 25,000 people in attendance for their model.^3^

While the Arbon model was created from a wide variety of events, it is best applied to the events based on those from which it was developed - events with greater than 25,000 people, which is much larger than any UCI World Cup race and similar more rural events. When applied to data from both the 2019 and 2021 races, the Arbon model yielded a result approximately 10% lower than the actual presenting value for both 2019 and 2021. There are likely several reasons for the under-estimated presentation rates. First, in the Arbon studies, the types of events included races and concerts where the majority of patient presentations were spectators. In this study, the majority of the presentations were adventure sport participants. This is likely due to the inherent dangers associated with downhill and cross-country mountain biking. The average member of the population in this study likely participated in more risky behavior than the average race or concert spectator in the Arbon study, which will be discussed further in the limitations section below.

In 2008, Hartman, et al retrospectively gathered data from 55 mass-gathering events (defined as > 1,000 people) and developed a classification system that stratifies events based on weather, attendance number and demographics, presence of alcohol, and crowd intentions to predict medical needs at mass-gathering events. Each event was analyzed based on the categories and given scores of zero to two with zero assigned to categories with minimal impact and two assigned to those with significant impact. Events with total scores greater than or equal to five were deemed “major,” scores of four were “intermediate,” and scores of three or below were “minor.” Examples of minor events include high school graduations and small concerts, intermediate events were collegiate basketball games, and major were collegiate football games and university graduations. They determined that minor events require one transport unit with an ALS provider and a BLS provider. Intermediate events should have several of both ALS and BLS providers and two transport units. Major events require multiple ALS and BLS providers, specialized equipment, and a physician presence to reduce the need for medical transport. While this paper does provide specific details about provider training recommended for these events, it does not predict the number of injuries expected or equipment needed to manage the anticipated injuries.^4^

Employing the Hartman model, the UCI World Cup events are classified as “intermediate,” with a recommended medical presence of both ALS and BLS personnel and two transport units. The 2021 event produced eight patient referrals to receiving medical facilities, though the majority of these patients were stable for transport via private vehicle, as shown in Table 3. However, based on the assessment, physician presence at the event prevented at least ten additional transports. Grange, et al demonstrated a nine percent reduction in patient transportations during a mass gathering at California Speedway (San Bernardino County, California USA) with a physician-led multidisciplinary team. The reduction noted in this cohort was 56%. These prevented transports included multiple laceration repairs and several cases of blunt abdominal trauma with reassuring initial assessments that were monitored with serial abdominal exams before return to competition. If physician skill had not been available on-site to treat these patients and prevent transport, local Emergency Medical Services (EMS) would have been needed for up to 18 transports. The Hartman team acknowledged this as well: “The presence of highly trained personnel did, however, reduce the number of patients who required transport, in many cases.”^4^

The closest receiving facility was a 45-minute one-way trip on mountainous rural roads. With limited local resources given the rural location, this would have taken already strained local EMS crews out of service for significant amounts of time. Additionally, many of the injuries were able to be treated by physicians on-site, allowing these professional athletes to return to competition immediately. One patient did require physician stabilization and air medical evacuation for poly-system traumatic injuries. The need for air transport services was not assessed in the Hartman study. If resources had been prepared based on the Hartman model prediction, these resources would not have been available. The authors feel that this is less likely due to inadequate prediction of the model but is likely secondary to the high-risk nature of this specific event. Additionally, the Hartman model was based on urban events such as collegiate football games which often occur in close proximity to tertiary care facilities which eliminates the need for helicopter EMS (HEMS) services.

The expanded model released in a mass-gathering event planning guide by the state of Minnesota in 2015, as mentioned above, does account for longer transport times, and had it been employed to predict medical staffing needed for the UCI event, physician presence would have been suggested.^8^ While similar events may classify as intermediate by assessment with the Hartman model, understanding the high-risk nature of competitive and often rural downhill and cross-country mountain bike racing is key to ensuring proper staffing. The authors recommend a dedicated EMS transport crew, HEMS crews in the immediate region, and physician presence to improve patient safety, promote safe return to racing, and prevent local community resources from becoming overwhelmed. If possible, physician certification should optimally include formal emergency medicine training and, if available, EMS/Disaster Medicine subspeciality education.

## Limitations

There were multiple limitations to this study. First, weather data were obtained retrospectively and therefore had to be based on highs/lows and averaged to be plugged in to predictive models. Future collection of data will be done on scene with verified technology. Second, both spectators and participants were included in the study. However, the medical clinic was not advertised openly in most highly traveled areas for spectators so it is reasonable to assume that a number of patients presented to local emergency departments without knowledge of services being provided at the event. These individuals were at risk of failure to capture. Third, rate of presentation was calculated based on riders participating and not laps on the course. A better method may be to observe how many individual laps were taken, as increased laps would likely increase rates of presentation. Additionally, the majority of patients were participants, which aligns with the risky nature of the activity. Since laps and riders are likely consistent between events, but spectators may vary widely based on event, it may be better suited to focus on participants only.

Additionally, there is subjectivity in determining how many points to assign to each variable when applying the Hartman model. For this study, classification was independently accounted for and subsequently agreed upon by each of the authors in an effort to limit this possible effect. For example, alcohol consumption is difficult to quantify and was potentially significant at this event for some spectators, but negligible for others. Additionally, alcohol consumption was much higher after daytime hours – during which the clinic was closed. If alcohol consumption had been deemed significant overall, the event would have been scored as a five, which would then classify the UCI race as a “major” event, requiring physician presence.

## Conclusions

Overall, the Arbon model provided a predicted patient presentation rate within reasonable error to allow for effective pre-event planning and resource allocation with only a four patient presentation difference from the actual data. A similar difference was seen for both the 2019 and 2021 events. Further studies of similarly-sized events may solidify this relationship, but studies of other non-cycling events will help demonstrate the usefulness of this model for small events. The high-risk nature of downhill and cross-country mountain biking may falsely increase the number of patient presentations compared to lower-risk, similarly sized events.

While the Arbon model under-predicted patient presentations, the Hartman model under-estimated resources needed due to the high-risk nature of downhill cycling. The events staffed by the authors required physician skill and air medical services to safely care for patients. Further evaluation of rural events will be needed to determine if there is a generalized need for physician presence at smaller events with inherently risky activities, or if this recurring cycling event is an outlier.

Finally, the data obtained from the 2019 UCI World Cup were a helpful source for determining resources needed and expected numbers of patient presentations for the 2021 event. Continuing to obtain data and analyze resources needed from these events will be essential in guiding medical care for future races.
